# The Gesellschaft für Medizinische Ausbildung (GMA) celebrates 40 Years of Education Research

**DOI:** 10.3205/zma001173

**Published:** 2018-05-15

**Authors:** Thomas Shiozawa-Bayer, Thorsten Schäfer

**Affiliations:** 1 Eberhard Karls Universität Tübingen, Institut für Klinische Anatomie und Zellanalytik, Tübingen, Germany; 2 Gesellschaft für Medizinische Ausbildung (GMA) e.V., Schriftführer, Erlangen, Germany; 3 Ruhr-Universität Bochum, Zentrum für Medizinische Lehre, Bochum, Germany; 4 Gesellschaft für Medizinische Ausbildung (GMA) e.V., Vorsitzender des Vorstandes, Erlangen, Germany

## Editorial

The Gesellschaft für Medizinische Ausbildung (GMA) will celebrate its 40th anniversary this year.

On April 22, 1978, at the initiative of the then AMEE president, Prof. Wolter, the Gesellschaft für Medizinische Ausbildung was founded as the German section of the Association for Medical Education in Europe (AMEE). Prof. Hans-Joachim Krämer, at the time IMPP president and Prof. Helmut Valentin, president of the Medizinischer Fakultätentag (MFT), belonged to the founding members of our association in that they recognized the need to combine German research and development for the purpose of medical education. Long-standing MFT president, Prof. Fritz Kemper later served on the GMA executive board, followed by Prof. Jörg-Dietrich Hoppe in 1982, chairman of the Marburger Bund and later president of the German Medical Association (Bundesärztekammer). Prof. Dietrich Habeck (Münster), who served as GMA president from 1981 to 1994, continued the close collaboration between GMA and MFT.

Six years after its founding GMA established it own academic journal entitled Medizinische Ausbildung. The journal was initially self-published under the editorship of Prof. Habeck with support from the Hans Neuffer Foundation. At the end of the 1980s and the beginning of the 1990s and at the prompting of the Robert Bosch Foundation, GMA members participated in the Murrhardter Circle which was tasked with defining the future profile of the medical profession and proposing suggestions for reforming university medical education, as had already been called for in 1986 by the Bundesrat. After the retirement of Prof. Habeck, Prof. Florian Eitel (Munich) was elected as the new GMA president at the members’ meeting in 1995. Prof. Habeck continued to head the editorial board of Medizinische Ausbildung and was appointed honorary president of the GMA in 1997. From 1998 to 2004 Medizinische Ausbildung was housed at the publisher Thieme Verlag Stuttgart. During this time Prof. Eitel also assumed the editorship. Even though the basic reforms to medical education did not take place at first, one major demand of the Murrhardter Circle was implemented in 1999: the “experimentation clause” (Experimentierklausel) was adopted in the 8th amendment to the German medical licensure act (Approbationsordnung) and for the first time model curricula were possible, such as the ones at Berlin’s Charité and the University of Witten-Herdecke.

In 2003 Prof. Eckhard Hahn was elected as the new president of GMA which grew substantially, more than doubling its members under his leadership. During this time the journal underwent modernization and has since been published by German Medical Science (GMS), an online platform belonging to DIMDI and ZBMed. As a consequence, the journal was changed to have an open access format. GMA’s efforts have been shaped by the new changes brought on by the amendments to the licensure regulations in 2002 and later by the Bologna Reform that affected higher education across Europe. The Directive “European Qualifications Framework for Lifelong Learning” (EQF-LLL) stemming from the Bologna and Lisbon Declarations made it necessary to introduce competency levels in medical education. In January 2009, the KMK committee for higher education (Kultusministerkonferenz) charged GMA, together with the MFT, to develop a framework for specialty qualification to be used in medical education. In the ensuing years the project committee, and later the steering committee, undertook the drafting of the National Catalogue of Competency-based Learning Objectives for Undergraduate Medical Education (NKLM), the content of which was significantly furthered by the Advisory Board (Beirat) and the GMA committees.

In 2011 Prof. Martin R. Fischer (Munich) assumed the presidency of GMA. During his tenure the NKLM was completed in 2017 and the National Catalogue of Competency-based Learning Objectives for Undergraduate Dentistry (KNLZ) was also drafted. After both catalogues were adopted at the Medizinischer Fakultätentag in 2015, implementation began—the next, large and ongoing reform to medical and dental education. In the meantime, policy makers have made their own plans for medical programs known in the Master Plan for Medical Education 2020. The 1955 German regulations governing licensure in dental medicine, however, still remain in critical need of reform.

Today, GMA unites more than 1,000 education researchers in Austria, Switzerland and Germany from a wide range of disciplines and professions into one academic and scientific society. The current president as of 2017 is Prof. Thorsten Schäfer of Bochum. The tasks for the coming year are diverse: medical schools are facing major changes with the new competency-based approach to curricula, assessments and, above all, state examinations. GMA perceived the importance of over-arching cooperation between the health professions in the area of education early on and has not only dedicated a wide-reaching special journal issue to the topic of interprofessionalism, but has also added an advisory member to its executive board. Digitalization in medicine and medical education is yet another issue to be confronted in higher education, as are scientifically anchored and innovative post-graduate programs and the transfer of the knowledge gained from research to medical education.

The authors are confident that the next 40 years will be just as exciting, if not more. *Ad multos annos*, GMA!

## Competing interests

The authors declare that they have no competing interests. 

